# CSO (Canadian Society of Otolaryngology – Head & Neck Surgery) position paper on return to Otolaryngology – Head & Neck Surgery Clinic Practice during the COVID-19 pandemic in Canada

**DOI:** 10.1186/s40463-020-00466-x

**Published:** 2020-10-26

**Authors:** Y. Chan, D. Angel, M. Aron, T. Hartl, S. P. Moubayed, K. A. Smith, D. D. Sommer, L. Sowerby, P. Spafford, D. Mertz, I. J. Witterick

**Affiliations:** 1 Department of Otolaryngology - Head & Neck Surgery, University of Toronto, Toronto, ON Canada; 2 Division of Otolaryngology - Head & Neck Surgery, Memorial University of Newfoundland, St. John’s, NL Canada; 3 Division of Otolaryngology - Head & Neck Surgery, Department of Surgery, University of British Columbia, Vancouver, BC Canada; 4 Division of Otolaryngology - Head & Neck Surgery, University of Montreal, Montreal, QC Canada; 5 Department of Otolaryngology - Head & Neck Surgery, University of Manitoba, Winnipeg, MB Canada; 6 Otolaryngology - Head & Neck Surgery Division, Department of Surgery, McMaster University, Hamilton, ON Canada; 7 Department of Otolaryngology - Head & Neck Surgery, Western University, London, ON Canada; 8 Division of Otolaryngology - Head and Neck Surgery, Department of Surgery, University of Saskatchewan, Saskatoon, SK Canada; 9 Division of Infectious Diseases, Department of Medicine, McMaster University, Hamilton, ON Canada

## Abstract

The novel Coronavirus (COVID-19) has created a worldwide deadly pandemic that has become a major public health challenge. All semi-urgent and elective medical care has come to a halt to conserve capacity to care for patients during this pandemic. As the numbers of COVID-19 cases decrease across Canada, our healthcare system also began to reopen various facilities and medical offices. The aim for this document is to compile the current evidence and provide expert consensus on the safe return to clinic practice in Otolaryngology – Head & Neck Surgery. These recommendations will also summarize general precaution principles and practical tips for office across Canada to optimize patient and provider safety. Risk assessment and patient selection are crucial to minimizing exposure to COVID-19. Controversial topics such as COVID-19 mode of transmission, duration of exposure, personal protective equipment, and aerosol-generating procedures will be analyzed and discussed. Practical solutions of pre-visit office preparation, front office and examination room set-up, and check out procedures are explored. Specific considerations for audiology, pediatric population, and high risk AGMPs are also addressed. Given that the literature surrounding COVID-19 is rapidly evolving, these guidelines will serve to start our specialty back into practice over the next weeks to months and they may change as we learn more about this disease.

## Introduction and rationale

Planning will be crucial to the success and sustainability of our transition to reopening our clinical practices. Ideally, the return to practice should be incremental and stepwise, incorporating virtual/telephone visits combined with in-person clinic visits. The aim of this document is to summarize the current evidence and provide expert consensus surrounding precautions for clinical care in the setting of the SARS-CoV-2 (COVID-19) pandemic. As well, these recommendations endeavour to provide general principles as well as practical tips for the reopening of Otolaryngology office practice to optimize patient and provider safety while providing a high level of patient care.

The literature surrounding COVID-19 is evolving rapidly. These guidelines will serve as a “starter” document to get our specialty back into practice initially over the next weeks to months. As the epidemiology of the disease changes, these recommendations may change. This text represents a living document that will be updated over time. Please see the online version (https://www.entcanada.org/news-events/covid-19-alerts/) for the most up to date information. If substantial changes in clinical information arise, this taskforce will endeavour to keep this information updated periodically.

The guidelines and recommendations of each applicable regional health authority should be respected and checked regularly for updates. The information in this document is meant to be an adjunct to local recommendations but not to supersede them.

### Methodology

This document represents a combination of expert opinion, practical knowledge, and the best available evidence. Given that the COVID-19 pandemic is rapidly evolving and the evidence is limited, this document represents a consensus of expert opinion. To identify the applicable literature, the following search terms were used in PubMed/Medline, Medscape, and Google Scholar: COVID-19, coronavirus, SARS-CoV-2, SARS, office reopening, practice reopening, plastic surgery, otolaryngology, and head and neck surgery. The date of the initial search was May 10, 2020 and the date of the final search was May 20, 2020 for the initial taskforce document and Jun 20, 2020 for the paper submission in order to capture as much information as possible for both documents. A rapid timeline was implemented to help make information available to otolaryngologists across Canada trying to define best practices for office reopening during a pandemic.

This document was prepared in a collaborative effort by all of the authors. An initial outline was developed via email and a shared online document for review. Two rounds of virtual round table discussions were held, 1 week apart, where each point was reviewed by all authors until a consensus was reached. Statements were modified and updated as needed to achieve consensus. If a consensus was not possible, no recommendation was made.

The authors were identified by the lead authors (YC, IJW), with an emphasis on representing all subspecialties within otolaryngology and both academic and community practices. Some authors are hospital-based, some have their own private offices in the community, and work in both private office and hospital-based clinics. An effort was made to provide representation from coast to coast within Canada as well. The affiliations outlined in the authors section annotates the location of each authors practices. The specialty and location of practice of each contributor is noted in the affiliations section for transparency.

### Definitions

- COVID-19: disease caused by the 2019 novel coronavirus
- SARS-CoV-2: severe acute respiratory syndrome coronavirus 2 virus
- Aerosol: abbreviation for “aero-solution”, a suspension of fine liquid or solid particles in a gas < 5 μm
- Droplet: small drop of liquid > 5 μm
- AGMP: aerosol generating medical procedure, for the purposes of this document, AGMPs include any procedure that has a reasonable potential to result in the production of aerosols of varying sizes including droplet nuclei. It is recognized that some higher risk AGMPs are more likely to induce larger quantity and longer duration aerosol/droplet production while others are less likely to do so
- HCP: health care provider
- PCRA: point of care risk assessment. This is to be conducted before every patient interaction and includes assessment of the task, the patient, and the environment. It is used for determination of control measures, appropriate actions, and PPE type.
- PPE: personal protective equipment
- Contact Precautions [1]:
  - Gloves and gowns for HCPs
- Droplet/Contact Precautions [1]:
  - Source control: mask on the patient
  - Mask, gown, eye protection (face shield or goggles), and gloves for HCPs
- Enhanced Droplet/Contact Precaution:
  - Source control: mask on the patient
  - Fit-tested N95 or higher-level respirator, gown, eye protection (face shield or goggles), and gloves for HCPs

## Risk assessment

### Prevalence COVID-19 in your community

One important consideration when increasing in-person visits during the COVID-19 pandemic is the current disease burden within your community and region. Loco-regional health authorities continue to track overall cases of confirmed COVID-19 infections, active cases, current hospitalizations, and mortality rates. These numbers will help guide clinicians, where decreasing numbers and low community spread may indicate it is safe to resume some in-person care and increasing numbers should trigger a significant reduction in routine in-person care. Moreover, testing should be available at a meaningful scale to confirm declining prevalence with decreasing incidence reflecting an actual decrease in disease prevalence rather than decrease in testing.

Beyond this, most health care resources are managed regionally or provincially. The authors would suggest considering the recommendations specific to your location as in-person visits are reintroduced to avoid unnecessary and potentially unsafe strains on local resources. For example, personal protective equipment (PPE) availability may affect the ability of some areas in Canada to ramp up in-person visits. Other regions have made recommendations about the division of in-person versus virtual care visits, with most regions recommending slow, progressive increases.

### Personal risk/comorbidity

The literature on COVID-19 suggests that personal comorbidities and characteristics can increase the risk of severe infection and mortality. Amongst other factors, advanced age, cardiovascular disease, and diabetes have been associated with an increased risk of death following COVID-19 infection. With this in mind, it is reasonable for physicians to consider their own comorbidities when defining their individual risk tolerance [2, 3]. To respect these individual variations, this task force will present options/recommendations for safe office practices and appropriate PPE.

However, these recommendations should be considered as “minimum reasonable PPE”, and providers should have the option to enhance their PPE, for example, with an N95 respirator, based on their point of care risk assessment (PCRA). However, supply chain concerns and sustainability should be also considered.

### Patient selection

As we move to increase in-person visits, it is important to consider that elective in-person visits should be limited to patients with a low risk of COVID-19 infection. Specifically, patients who have no signs or symptoms of COVID-19 for the last 14 days, no unwell contacts, no recent travel to a jurisdiction with a higher prevalence, and no contact with COVID-19 positive patients - in the setting of low community spread. In areas with high community spread or increasing infection rates, in-person visits should be limited to essential visits only. The recommendations in this document do not apply in the latter scenario.

It may also be reasonable to consider having the physician screen and vet the need for in-person appointments (versus virtual/telephone visit) for appropriateness at this time.

When considering procedures in low risk patients, another important factor is patient cooperation or tolerance. It may be reasonable to defer elective in office procedures (endoscopy, cerumen debridement) in patients who are less tolerant (high gag reflex, lower pain or discomfort tolerances) for the safety of other patients and health care workers.

## Controversies

### Mode of COVID-19 transmission

The primary mode of transmission for COVID-19 is believed to be droplet /contact [4]. However, there is some controversy on whether aerosol spread of COVID-19 is significant for disease transmission. The World Health Organization (WHO) as well as Canadian Public Health agencies currently recommend droplet/contact precautions for HCPs caring for COVID-19 patients and additional airborne precautions for AGMPs according to PCRA.

### High vs low risk AGMPs in the office

Talking, coughing, sneezing, and even breathing can generate droplets [5] and aerosols [6], but there is insufficient evidence at this point that virus transmission through aerosols in this setting are relevant, neither for COVID-19.

A number of medical procedures are potentially aerosol-generating but evidence for the creation of aerosols and the burden of viable infectious particles within the aerosols are not well studied. A systematic review by Tran et al. examined the risks of AGMP associated transmission of acute respiratory infections in HCPs and found that tracheal intubation and tracheotomy were associated with transmission of the SARS-CoV-1 virus during the 2003 outbreak, while bronchoscopy, nasogastric (NG) tube insertion, and suctioning of body fluids were not [7]. Given the association of viral transmission, tracheal intubation and tracheotomy are considered high risk AGMPs.

Flexible nasopharyngolaryngoscopy (NPL) represents an area of challenge and confusion for our specialty as it is not consistently recognized as an AGMP. So, is flexible NPL an AGMP or not? Some of our provincial authorities as well as the CDC and WHO do not recognize it as an AGMP. Nasopharyngolaryngoscopy was initially thought to be aerosol generating only when a sneeze or cough was induced [8]; however, there is new evidence that nasal endoscopy produces airborne aerosol with both rigid and flexible endoscopes [9]. Given its similar invasiveness to NG tube insertion and that NG tube insertion was not found to be a high risk AGMP [7], it is reasonable to extrapolate that flexible NPL is likely to be a low risk procedure, even if it generates aerosols.

Similarly, there is also no evidence that sneezing or coughing induced by nasopharyngeal specimen collection leads to increased risk of transmission of COVID-19, justifying recommendations for HCPs to use contact/droplet precautions when performing this task. Again, this is a procedure similar to flexible NPL in its trajectory and risk of inducing coughing or sneezing. Arguably, flexible NPL is even less traumatic than nasopharyngeal swabbing given that it is done under direct visualization.

**Fig. 1:**
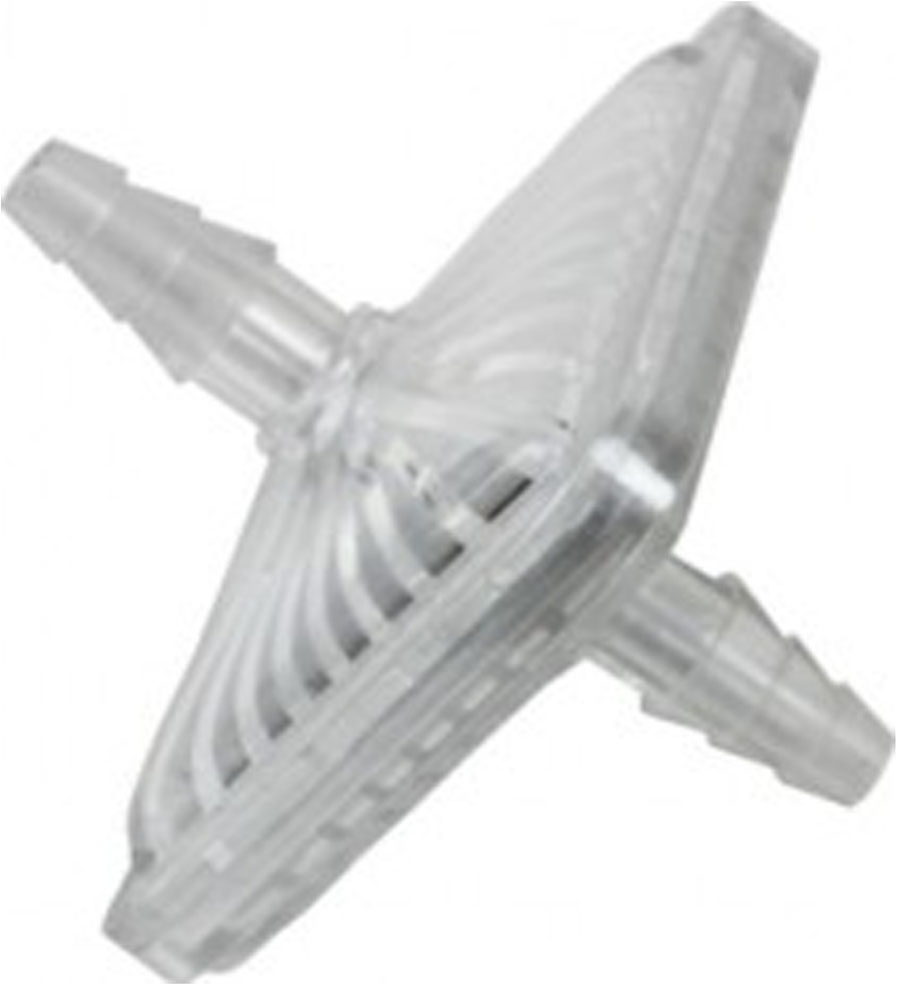
Hydrophobic suction filter

Suctioning of middle ear fluid is another area of controversy during the COVID-19 pandemic. In viral URTI, previous studies have demonstrated that viruses detected in the nasopharynx can also be detected in middle ear fluids [10]. Extrapolating from this, but in the absence of direct evidence, middle ear and mastoid fluid should be treated as potentially coronavirus-containing in infected patients. Although there is evidence that open tracheal suctioning especially in mechanically ventilated patients may generate aerosols [11], there is no convincing evidence that suctioning in general results in aerosol generation. In fact, a recent cadaveric study demonstrated absence of aerosol generation with nasal suctioning using a 10F Frazier suction [9]. Furthermore, other work has shown that suctioning has the potential to reduce aerosol spread from potential AGMPs [12]. This taskforce would consider nasal suctioning as a low risk AGMP and ear suctioning a non-AGMP or low risk AGMP in the case of tympanic membrane perforation or incision (myringotomy). It has been demonstrated that a stand-alone suction machine in most standard otolaryngology cabinets disperses aerosols from the suction into the air, thus a suction filter (see Fig. 1) is strongly recommended [13].

Given this evolving literature, patients should wear a mask throughout their in-person visit to minimize any potential droplets or aerosols that may be generated throughout their visit. It is also recommend that, at a minimum, the patient and the physician should be wearing a surgical mask for both source control and personal protection during endoscopy and microdebridement of the ear canal at this time.

### Is air cleaning necessary?

To determine if air cleaning is necessary following otolaryngology office visits which may include NPL and other AGMPs, we need to examine the evidence of COVID-19 viral particles that may be present in the air after exposure to a COVID-19 positive patient. A recent experimental study evaluated the persistence of COVID-19 viral particles and found that under artificial conditions, aerosols generated by a three-jet Collison nebulizer and fed into a Goldberg drum lasted up to 3 h in the air [14]. Caution must be used to interpret this data as a powered generator does not simulate human cough condition or any AGMPs performed in clinical settings.

Furthermore, a report from Hong Kong by Cheng et al. where air samples were taken from a COVID-19 positive symptomatic patient 10 cm away from the patient’s chin while performing normal breathing, deep breathing, speaking “1,2,3” continuously, and coughing continuously with and without mask use did not reveal any detectable SARS-CoV-2 RNA [15]. This anecdotal evidence needs to be taken in the context of potential large variability in individuals’ propensity for aerosol generation - while some patients produce little to no aerosol, others are considered high-producers. Edwards et al. sampled exhaled bioaerosols from 11 subjects and found that 6 of these produced over 99% of the bioaerosols measured [16]. Similarly, Asadi et al. used an aerodynamic particle sizer to demonstrate that 8 of 40 individuals tested produced an order of magnitude greater aerosol particles during speech production than their counterparts [17]. These ‘super-emitters’ may contribute to the phenomenon of superspreading of aerosolized infection, however it remains unproven if this type of transmission is applicable to the current pandemic.

In-room air cleaning units are effective in protecting HCPs from airborne pathogens such as measles and tuberculosis. Currently, there is insufficient evidence that SARS-CoV-2 is an airborne pathogen. Free aerosol generation is minimized in our encounter with the patient by having them wear a mask [18] and limiting encounter duration. In such a setting, air purifiers or air exchangers are not mandatory. If there is concern about significant aerosols being generated during a visit, air cleaning may be beneficial and should be considered as an option. There is limited evidence on which air cleaning devices should be considered at this time.

### Duration of exposure

There is a paucity of information on the transmission of COVID-19 in healthcare settings in terms of duration of exposure. A recent study in the CDC’s Morbidity and Mortality Report described the exposure to COVID-19 among HCPs who did and did not acquire COVID-19 infection [19]. HCPs who contracted COVID-19 had a longer duration of exposure compared to those who did not develop the infection. The median estimated exposure duration in COVID-19 positive HCPs was higher (120 min) compared to COVID-19 negative HCP (25 min) (*p* = 0.06). The median duration of exposure during AGMPs was also higher among HCP with COVID-19 (95 min) than among those without COVID-19 (0 min) (*p* = 0.13). At the time, COVID-19 was not suspected, HCPs were unprotected except for gloves and surgical masks in some instances. No N95 respirator or eye protection was used for AGMPs during exposure. There was also no patient source control measure such as wearing a mask.

Ng et al. reported that in a case where 41 HCPs had contact with a COVID-19 positive patient (unknown at the time) during an AGMP of at least 10 min at a distance less than 2 m wearing surgical masks (some with N95) and gloves did not contract COVID-19 infection [20]. The CDC currently uses 15 min as an operational definition for prolonged exposure, but data to justify this arbitrary cut-off is limited [21].

From current available evidence, transmission of COVID-19 requires prolonged and unprotected contact with an infected individual. Hence, we recommend contact/droplet precautions for HCPs during otolaryngology outpatient encounters given that the duration of contact with these patients is limited, and to consider masking the patient whenever possible in particular in small areas when physical distancing cannot be adhered to. We suggest performing all procedures efficiently and limiting time with the patient both pre and post procedure. As with all encounters, PCRA is performed at each encounter and PPE may be modified accordingly.

### Room decontamination post visit

Environmental contamination has been implicated as a route of transmission. In a study from Singapore examining areas with extensive exposure to actively viral shedding COVID-19 patients found that post cleaning samples were negative for SARS-CoV-2 virus following twice daily cleaning [22]. This indicates that routinely wiping down high contact areas will suffice, and should be done between patients (patient chair, door handle, etc.). Please see reference [23] for a list of approved cleaning products effective for COVID-19 [23].

## Pre-visit considerations

A number of pre-visit issues should be considered prior to having in-person visit appointments in order to prepare the office optimally (See Appendices 1 and 2).

### Office staff COVID-19 education

The office staff should become familiar with and should be up to date on the epidemiology of COVID-19 in your practice catchment area. The office personnel should practice physical distancing, keep 2 m distance from others whenever possible, practice hand hygiene (soap and water or alcohol-based (minimum (70% alcohol) hand sanitizer), avoid touching their face, and cover their cough or sneeze with elbow or tissue. It should be made clear to everyone that they should stay at home if they are sick. Frequently touched surfaces should be cleaned and disinfected twice daily. The staff should have a face mask or face covering if physical distancing is not possible in the office.

### Office preparedness [24]

The office should prepare for office staff illness/absences by cross-training staff for all essential office functions to ensure coverage when personnel are sick. The office should establish proper office and medical cleaning routines and the entire staff should be familiar with these protocols. Where possible, all doors along the patient path from entrance through office to exit could be kept open to minimize contact with door handles.

#### Clinic/waiting room considerations

The patient waiting area will need to be modified to help mitigate risks of SARS-CoV-2 transmission. Waiting room chairs should be spaced 2 m apart whenever possible. To protect patients and office personnel, a front desk barrier (sneeze guard) can be considered. It is suggested that magazines, hand outs, non-essential objects be removed to decrease chances of contamination. Handling of clipboards and pens in patient care areas should be minimized. One may consider having dedicated pens for patient use. At the front desk, office staff should minimize sharing of items such as computer keyboard, mouse, and stationary. No touch trash bins with disposable linings can be considered to decrease contact surface. Hand sanitizers should be readily available for patients and front desk staff. It is helpful to have signage to remind patients of respiratory hygiene, cough etiquette, arrival instructions, COVID-19 screening questions. There should be signs to indicate clearly where people should sit/stand while waiting to be able to respect physical distancing (e.g. floor decals). If multiple doors leading into the clinic are available, one may take advantage to create separate entrance and exit for patients if possible.

Front staff is to advise patients before their visit that they should wear face mask or covering in the waiting area and during clinic visit whenever feasible. Patients should be instructed to arrive on time (not early), to manage waiting room crowding. Patients may be able to wait outside of the building or in their vehicle if they arrive too early and receive a phone call to enter only just prior to appointment time to reduce time and crowding in the waiting room. To minimize waiting room congestion, patients should be advised that they are to arrive unaccompanied unless absolutely necessary if they require a caregiver or translator.

#### Exam room considerations

There are many measures that one can use to minimize environmental contamination such as decluttering the examination room space by removing hand-outs, models, and exposed equipment. Hand-outs and models can be stored in drawers and cupboards and equipment as well as instruments should be covered. All the instruments required for the examination should be available on the counter to avoid opening of the drawers during procedures. Dirty instruments and scope should be transported using covered bins as much as possible. “Used reusable PPE” (goggles/masks) container should be covered. Hand sanitizer should be readily available and accessible in the examination room. High touchpoint areas should be cleared after each patient using approved cleaners and wipes [23]. A hydrophobic disposable suction filter is recommended on the suction machine to decrease particulate recirculation in the air after suction use (see Fig. 1).

A plastic cover on keyboard will allow it to be wiped and cleaned more easily. Barriers (plexiglass) may be considered for difficult to clean/protect areas (e.g. computers, electronics, endoscopy towers). Air exchange in clinic rooms is heavily variable and depends on dimensions of room and HVAC (Heating, ventilation, and air conditioning) characteristics. It is unclear if or which settings may benefit significantly from HEPA (High efficiency particulate air) filtration and this may be further discussed with an HVAC specialist at the provider’s discretion.

### Referral triage: virtual vs in-person visit

Establishing the appropriate number of patients scheduled per hour is prudent to allow for increased time to account for patient flow, encounter, and room cleaning. Starting conservatively and ramping up as appropriate over 3–6 weeks will help ensure compliance and limit waiting room overcrowding. There should be a system in place (usually by MD) to triage referral to determine if the patient requires an in-person visit or a virtual/phone visit first.

The physician may consider taking a history via a virtual/phone visit first and then scheduling a clinic visit as appropriate. This is drastically limit in-person contact time to the physical examination and treatment plan formulation. A Head & Neck cancer risk calculator http://www.orlhealth.com/risk-calculator-2.html may be useful as a screening tool [25] to determine if the patient is high risk for cancer and how urgently he/she needs to be assessed in person. Through virtual/telephone visits, investigations can be ordered with a trial of medications started. Pre-visit imaging (e.g. CT for patients with specific sino-nasal complaints or U/S for thyroid nodule) after the virtual/telephone consultation can help facilitate the patient care.

Virtual/telephone visits may be considered for the following groups of patients such as new patient consultation for history first, follow up for review of test results, follow up for some post operative patients, follow up for patients with certain chronic disorders, medication refills, review of preoperative questions and patients with significant comorbidities in order to limit their exposure to other patients and out of home settings. In-person visit should be reserved for patients requiring physical examination and therapeutic or diagnostic interventions.

### Clinic schedule considerations

Virtual visits can be scheduled in between in-person encounters to allow for cleaning and minimize patient flow in waiting room. If multiple physicians share a practice, it is important to consider staggering patient appointments to avoid overcrowding at front desk and waiting area.

### Pre-appointment phone call [26]

#### Create a script (see Appendix 3)

Prior to an in-person visit, a pre-appointment phone call is important to determine the patient’s suitability to come into the office/clinic. Ideally these should be done within 24–48 h of the appointment. A printed script available for the administrative assistant may be helpful. COVID-19 screening questions (see Appendix 4) should be included in the telephone call. If patient fails screening, his/her appointment may be changed to virtual visit or reschedule his/her in-person visit.

It is also suggested that patients be given detailed instructions about how to safely attend their appointment in person, either via verbal communication from the office staff or with a form sent to the patient by email or mail. Patients should be instructed to bring and wear a mask to the appointment from the time they enter to the time they leave the clinic. There should also be clear instructions about where patients should wait if they arrive early for their appointment. This will depend on your office setup and how your space and setup allow you to respect physical distancing and can include marked physically-distanced spots (e.g. floor decals) in the waiting room or hallway or perhaps even having patients wait outside the clinic or in their cars until the time of their appointment. It is important to advise patient to arrive unaccompanied if possible unless they need caregiver or translator to reduce waiting room crowding.

## Patient visit

### Patient check in

Any necessary identifying information from the patient can be collected over the phone at the time of appointment confirmation. Alternatively, or additionally, patients can be sent questionnaires and/or forms (ex: fillable PDFs) by email to be filled out and returned digitally before their appointment. Completing these steps before the in-person visit helps minimize time of contact between office staff and patients as well avoiding sharing of contact points by passing papers back and forth between them.

Upon arrival to the office, patients should be instructed to sanitize their hands at a hand sanitizing station set up at the clinic entrance. If not already wearing a mask, patients should be asked to put one. A surgical mask may be provided to any patient who did not bring one to their visit in order to help protect themselves, the office staff as well as other patients in the waiting room. If confirmation of identity or health card number is needed upon check in, patients can be asked to hold up their card to the glass barrier so as, once again, to minimize physical exchange between patients and staff. Patients should be reminded of physical distancing and assigned holding areas in the waiting area at check in.

At check in, patients should be screened for COVID-19 symptoms, once again, with suggestion of a phone assessment to replace the in-person assessment for patients who screen positive. Routine patient temperature checks are currently recommended against by this taskforce as isolated fever is an uncommon presentation of COVID-19 infection and a normal temperature reading may give health providers a false sense of security.

### Patient-physician encounter (summarized in Table 1)

#### Donning PPE (see appendix for infographics)

PPE should be kept and donned in a designated clean space outside the examining room. The CDC has published instructions for the proper donning of PPE and has also provided visual aids that can be printed and posted in your office PPE donning space for medical staff to use as reference [28].

**Table 1:** Summary of AGMPs, Risks, and Appropriate PPE Considerations

Encounter Type	Possible Aerosol Generation	Risk	Patient Mask	Physician PPE^a^	Special Considerations
History	Minimal	Low	Yes	Surgical Mask, Eye Protection^b^	Consider virtual history to minimize in-person time Maintain physical distancing in person
Physical Exam	Minimal	Low	Yes (if possible)	Surgical Mask, Eye protection^b^, Gloves	Time with mask off (of patient) should be minimized where possible
Transtympanic Injection	Minimal	Low	Yes	Surgical Mask, Eye Protection^b^, Gloves	Beware Arnold’s reflex Consider local anesthesia/blocks to reduce coughing for extensive debridement Use of microscope may be hindered by eye protection
Tympanostomy Tube Placement	Minimal	Low	Yes		
Mastoid/EAC Debridement	Minimal	Low	Yes		
Cauterization Epistaxis	Minimal	Low	Yes	Surgical Mask, Eye Protection, Gloves, Gown^b^	Consider chemical cautery over electrocautery
Rigid Nasal Endoscopy	Moderate	Low	Yes		Avoid powered aerosolized anesthetic sprays Pull mask down to expose nostril or modify mask, keep mouth covered Consider video system over direct visualization to increase distance from patient
Flexible Nasopharyngo-laryngoscopy	Moderate	Low	Yes		
Tracheotomy/Laryngectomy Patient	High	High	Not applicable	N95 respirator or Surgical Mask Eye Protection, Gloves, Gown	Unable to cover cough Consider N95 mask, particularly if tube being manipulated or suctioning [27] performed
Rigid Laryngoscopy	No consensus	–	No consensus	No consensus	Consider flexible nasopharyngolaryngoscopy as an alternative
Aesthetic Procedures	Minimal	Low	Yes (if possible)	Surgical Mask, Eye Protection, Gloves, Gown^b^	Consider availability of appropriate PPE Dependent on local health authority recommendations

^a^minimum recommended personal protective equipment, ^b^optional PPE, at physician discretion

#### History (low risk AGMP)

When possible, medical history can be taken over the phone during an initial virtual visit in order to minimize the amount of time spent interacting in person during the clinic visit. This is particularly important in cases where you may expect the history portion to require a prolonged discussion, for example, patients with dizziness. When taking a history in-person, attempts should be made to maintain physical distancing as much as possible, and masks should be worn by the physician and the patient at all times and minimizing face-to-face positioning (e.g. sitting side-to-side) to help avoid exposure to droplets or aerosols during the verbal exchange.

#### Nasopharyngolaryngoscopy / rigid nasal endoscopy with or without suction (low risk AGMP)

During the pandemic, it is prudent to determine if endoscopic examination is necessary or if it can be delayed. Surgical mask (consider N95 if higher prevalence or surgeon risk tolerance as per PCRA), eye protection (face shield/goggles/visor), gloves, and gown (optional) should be worn for this procedure. Powered application of aerosol anesthetic and decongestants are discouraged in favour of hand-held spray or lidocaine/decongestant pledget. Another option is to ask patients to pre-spray decongestant into their nasal cavities prior to visit.

A modified face mask [8] on the patient or simply lowering the face mask below the patient’s nose are acceptable options for scope access to nares. Instructing the patient to warn you if he/she needs to sneeze so you can remove the endoscope in order for him/her to sneeze into the elbow or put the back over the nose is a good way to minimize aerosols. A useful tip is to try pinching the nose at the rhinion or pressing firmly on the upper lip to abort the sneeze. Another option is to use a pediatric scope which is less likely to irritate the nasal mucosa than the adult scope due to the smaller diameter.

If available, a video system rather than direct visualization through endoscope eyepiece may be considered to keep examiner’s face away from patient during endoscopy. Mobile endoscopic adapters are a potentially useful and affordable alternative to a video system and allow the operator to increase the space between the patient and the physician (e.g. SMARTScope®, Karl Storz; Save my Scope®; Endoscope-i®).

#### Microdebridement external auditory canal or mastoid cavity (low risk AGMP)

A hydrophobic suction filter is recommended for the suction machine to help eliminate recirculation of particles in the air (see Fig. 1). Surgical mask and gloves (+/− eye protection (face shield/goggles/visor) are suggested for suctioning with no mucosal exposure. We also suggest a mask on the patient for source control. It is important to be aware of the risk of cough with EAC debridement because of Arnold’s reflex and warn the patient of this possibility and ask patient to notify you if he/she feels like he/she will cough. If continued debridement is necessary in a patient with sensitive cough reflex, it may be prudent to consider local anesthesia in EAC to complete the debridement.

#### Intratympanic injection/placement of tympanostomy tube (low risk AGMP)

For low risk AGMPs, it is suggested that surgical mask, gloves, +/−eye protection (face shield/goggles/visor) with a mask on patient will be sufficient. One may consider inserting a ventilation tube and instructing patient to self-administer medication at home via external canal (prescribed intratympanic solution) rather than repeated visits to the office to help decrease exposure.

#### Cauterization for epistaxis (low risk AGMP)

The cauterization for epistaxis is also considered a low risk AGMP. A surgical mask (consider N95 if higher prevalence or surgeon preference), eye protection (face shield/goggles/visor), gloves and gown are adequate protection for such procedure. Again, patient should wear a mask and the masked is to be pulled down to expose the nares. Topical anesthesia and decongestant are to be used via a pledget or manual spray bottle. Chemical cautery over electrocautery should be used given possible aerosol generation with electrocautery.

#### Tracheotomy or laryngectomy patients [29] (high risk)

Tracheotomy or laryngectomy tube change and suctioning are considered high risk given the direct communication with the respiratory tract. The provider should consider N95 respirator, gown, gloves, eye protection (googles/visor/face shield) for these procedures. Open tracheal suctioning should also be avoided if possible.

#### Aesthetic facial procedures [30]

Aesthetic facial procedures performed by Otolaryngologists-Head and Neck Surgeons in the office include but are not limited to botulinum toxin injections, dermal fillers, platelet-rich plasma injections, energy-based procedures on the face and neck (such as lasers and radiofrequency devices), non-invasive and invasive skin-treatment procedures (e.g. peels, microneedling, etc.). Resuming elective office procedures in aesthetic medicine should be dependent on local health authority recommendations as well as availability of appropriate PPEs.

Consolidating multiple patient treatments into a single treatment room and visit in order to minimize patient and practitioner exposure should be considered. All necessary supplies and equipment should be prepared and set out prior to bringing the patient into the treatment room.

Topical anesthetic agents (such as topical benzocaine/lidocaine/tetracaine ointment and others) should be applied in the same room as the ensuing treatment to minimize patient movement.

For injectables such as dermal fillers and botulinum toxin, although actual treatment duration is of only a few minutes, proximity to the patient’s nose and mouth during such procedures create exposure risk. The use of a surgical mask, gown, safety goggles, and gloves for the provider is suggested. A mask should be worn by the patient as long as it is safely possible to do so without preventing the actual procedure. Staff personnel in the treatment room should be reduced unless necessary. If staff assistance is required, similar PPE is recommended. Intraprocedural discussion by provider and patient should be minimized.

#### Special considerations for pediatric patients [31]

Pediatric outpatient visits are a current area of controversy in the Otolaryngology clinic setting. Evidence suggests that this population is more likely to be asymptomatic carriers, and less likely to adhere to office or exam protocols e.g. always arrive accompanied, frequently “screen positive” due to nonspecific URTI symptoms, and most importantly, be non-compliant with mask use. As such, a PCRA is suggested to be conducted for each pediatric patient considering factors such as disease severity, assessment of community disease prevalence, necessity of in-depth examination (e.g. use of tongue depressor, scope), cooperative vs uncooperative patient, treatment options, likelihood of aerosol generation and thus risk to HCP. Other tips may include placing a mask on patients over 2 years of age as tolerated and asking caregivers to prevent young patients from touching office equipment/surroundings.

### Audiology assessment

Audiology recommendations presume that staff and patient screening, hand hygiene, social distancing and mask wearing precautions (audiologist and patient) are adhered to as described above. Similarly, staff should consider remote/virtual assessments either prior to or instead of visits e.g. initial assessment/screening, hearing aid support as appropriate.

Recommendation for audiologist to wear mask, gloves and eye protection when placing equipment on patient or otherwise near patient/caregiver. Hand hygiene should be employed by audiologist when moving between sound booth and audiology equipment. Similarly, patients are to perform hand hygiene before and after testing as they will be interacting with equipment.

Equipment and other patient contact areas should be stratified into high risk (e.g. door handles, equipment with frequent contact such as patient response button, bone conductor) and low risk (e.g. walls) [32]. Higher risk surfaces should be cleaned/wiped with appropriate techniques between use, while lower risk surfaces may be addressed once daily.

HVAC concerns are somewhat similar to those discussed above for clinic rooms. Specific issues for audiology booths include relatively quick air exchange for booths (e.g.10 min for small booth, 15 min for larger booths, assuming they are connected to buildings’ HVAC systems), as well as consideration of noise pollution if HEPA filtration units are employed. At this juncture, there is insufficient evidence to recommend HEPA filtration and a specified waiting period between patients.

### Patient check out

During patient check out, physical distancing should be maintained at the front desk. The office may consider omitting patients stopping at the front desk upon completion of the visit to eliminate crowding of the waiting area. Electronic medical record (EMR)-based appointment renewal may be employed. Instructions for investigations that need to be ordered can be communicated to the administrative assistants via EMR tasks.

## Post-visit considerations

### Soiled medical equipment processing

Routine instrument cleaning using the approved processes in place is advised. It is optional to immerse reusable instruments in a container with soapy water immediately after use and transfer to device processing when the container is full or at the end of the day. The entire endoscope and light cable as well as battery pack are required to be cleaned since the handle may also be contaminated [33].

## PPE doffing procedures

The proper doffing of PPE procedure is summarized pictorially by CDC [28]. This may be the most important segment of the precautions we take as a health care team. First, there should be clear instructions of the post doffing procedure in the doffing area. All personnel should doff in the exam room itself. There should be adequate space to perform all manoeuvres including dedicated space to wipe down eyewear. Hand sanitizer which will be used frequently, as well as wipes, are to be accessible nearby. Gloves need to be safely disposed of after each patient.

Disposal versus cleaning and re-use of masks and gowns depend on numerous factors including risk tolerance, PPE availability, and especially concern about contamination which includes both the specific procedure performed and the extent of a potential soiling event. Finally, office surfaces, exposed objects, and contaminated eye protection should be cleaned with approved cleaner or wipes [23].

## Conclusions

In summary, our document collated current available evidence and presented the best practices for safe modifications Otolaryngologists can adapt in the clinic setting in order to provide optimal patient care during the era of COVID-19. Issues discussed in detail include risk assessment, patient selection, mode of COVID-19 transmission, duration of exposure, and personal protective equipment. Clinic and front office readiness concerns were also addressed through practical tips. The recommendations will serve to guide our specialty back into practice as more is known about COVID-19.

### Supplementary information

**Additional file 1.** Appendix 5: Sample signage for office use.

## Data Availability

Not applicable.

## Appendix 1

### Checklist of Possible Items to be Implemented

- ❏ Order clinic supplies
- ❏ Create pre-visit call script
- ❏ Create signage for office
- ❏ Reorganize front office
- ❏ Remove exam room chairs as appropriate
- ❏ Declutter waiting room, front desk, and exam rooms
- ❏ Cover keyboards, instruments, dirty trays, used PPE containers
- ❏ Train staff on PPE
- ❏ Train staff on new office protocols

## Appendix 2

### Checklist of Possible Items to be Purchased (pending availability)

- ❏ Plexiglass barrier (sneeze guard)
- ❏ Trash bins with disposable liners (foot activated)
- ❏ Surgical masks / N95 respirators
- ❏ Gloves
- ❏ Gowns
- ❏ Face shield /goggles/visors
- ❏ Hand sanitizer
- ❏ Approved cleaning wipes
- ❏ Minimum 70% Alcohol cleaning/disinfectant solution
- ❏ Instrument/scope transport containers
- ❏ Instrument containers with lids
- ❏ Plastic “dirty PPE” container
- ❏ Suction filter
- ❏ Keyboard cover
- ❏ Physical distancing floor decal
- ❏ Signage specific to COVID-19 and office protocols (see Additional file 1: Appendix 5)

## Appendix 3

### Possible script for Pre-Visit Telephone Call

**ENGLISH**

May I speak to [name of patient with scheduled visit]? I am calling from [Dr X’s office] with regard to your appointment scheduled for [date/time]. Given the recent COVID-19 outbreak, I am calling to ask a few questions in connection with your scheduled appointment. We are asking the same questions to all patients in preparation for the visit.

Administer Pre-COVID-19 screening questionnaire (Appendix 4).

If you can, please come by yourself unless you need a caregiver or translator. Please bring and wear a mask or face covering. Please check-in at the front desk and we may ask you to wait in your car to be called for your appointment after your check-in. Unless you hear otherwise from us, we look forward to seeing you at your appointment on [date/time].

**FRENCH**

*Est-ce que je peux parler avec [nom du patient]? J’appelle du bureau du docteur(e) [nom du médecin] par rapport à votre rendez-vous du [date/heure]. Compte-tenu de la situation de la COVID-19, j’aimerais vous poser des questions avant votre rendez-vous. On pose les mêmes questions à tous les patients en préparation aux rendez-vous.*

*Questionnaire Pre-COVID-19 (**Appendix 4**).*

*Si vous pouvez, venez seul(e), à moins d’avoir besoin d’aide ou de traduction. Amenez un masque ou un couvre-visage. Enregistrez-vous à l’accueil et il est possible qu’on vous demande d’attendre dans votre auto avant d’être appelé à votre tour. Nous vous attendons pour votre rendez-vous du [date/heure].*

## Appendix 4

### Pre-Visit COVID-19 Screening Form for All Patients [34]

**Table:**

Question		
1. Are you coming from a. home b. hospital c. long term care		
2. Have you had any of the following symptoms BEGIN in the last 14 days?	YES	NO
­ fever		
­ chills		
­ cough		
­ shortness of breath or breathing difficulties		
­ runny nose or nasal congestion		
­ joint or muscle pain		
­ new headache		
­ recent onset of loss of smell or taste		
­ weakness, exhaustion		
­ new onset diarrhea, nausea, vomiting		
­ pink eye		
­ any other symptoms or illness		
3. Have you been in contact with, or close to anyone who has the coronavirus in the last 14 days?		
4. Have you been in contact with, or close to someone who has been sick in the last 14 days (such as a cold, pneumonia, etc.), in absence of negative COVID-19 test.		
5. Have you travelled in the last 2 weeks? If so, where?		
6. Do you work in a high-risk facility and have you been tested positive for COVID-19? If so, when? *		

*Question 6: (case by case decision)

**FRENCH VERSION**

**Table:**

Question		
1. D’où venez vous? a. domicile b. hôpital c. CHSLD		
2. Avez-vous eu le symptôme suivant qui a DÉBUTÉ au courant des 14 derniers jours?	YES	NO
­ fièvre		
­ frissons		
­ toux		
­ difficulté à respirer		
­ écoulement nasal ou congestion nasale		
­ douleurs musculaires ou aux articulaires		
­ nouveau mal de tête		
­ perte du goût ou de l’odorat subite		
­ faiblesse, épuisement		
­ diarrhées, nausées, vomissements d’appartition soudaine		
­ yeux rouges		
­ tout autre symptôme ou maladie		
3. Avez-vous été proche ou en contact avec quelqu’un qui a eu le coronavirus dans les 14 derniers jours?		
4. Avez-vous été proche ou en contact avec quelqu’un de malade dans les 14 derniers jours (rhume, pneumonie, etc.), et vous n’avez pas eu de test négatif à la COVID-19?		
5. Avez-vous voyagé dans les deux dernières semaines? Si oui, où?		
6. Travaillez-vous dans un établissement à haut risque? Et si oui avez-vous été testé positif pour la COVID-19? Si oui, quand? *		

*Question 6: (décision cas par cas)

## Abbreviations

COVID-19: Disease caused by the 2019 novel coronavirus
SARS-CoV-2: Severe acute respiratory syndrome coronavirus 2 virus
Aerosol: Abbreviation for “aero-solution”
AGMP: Aerosol generating medical procedure
HCP: Health care provider
PCRA: Point of care risk assessment
PPE: Personal protective equipment
