# Supplementary material for: CSO (Canadian Society of Otolaryngology – Head & Neck Surgery) position paper on return to Otolaryngology – Head & Neck Surgery Clinic Practice during the COVID-19 pandemic in Canada
Source: J Otolaryngol Head Neck Surg. 2020 Oct 26;49:76. doi: 10.1186/s40463-020-00466-x (PMC7586368; doi:10.1186/s40463-020-00466-x)
Supplement: Supplementary file 1 — Additional file 1. Appendix 5: Sample signage for office use. [file 40463_2020_466_MOESM1_ESM.pptx]

## Slide 1
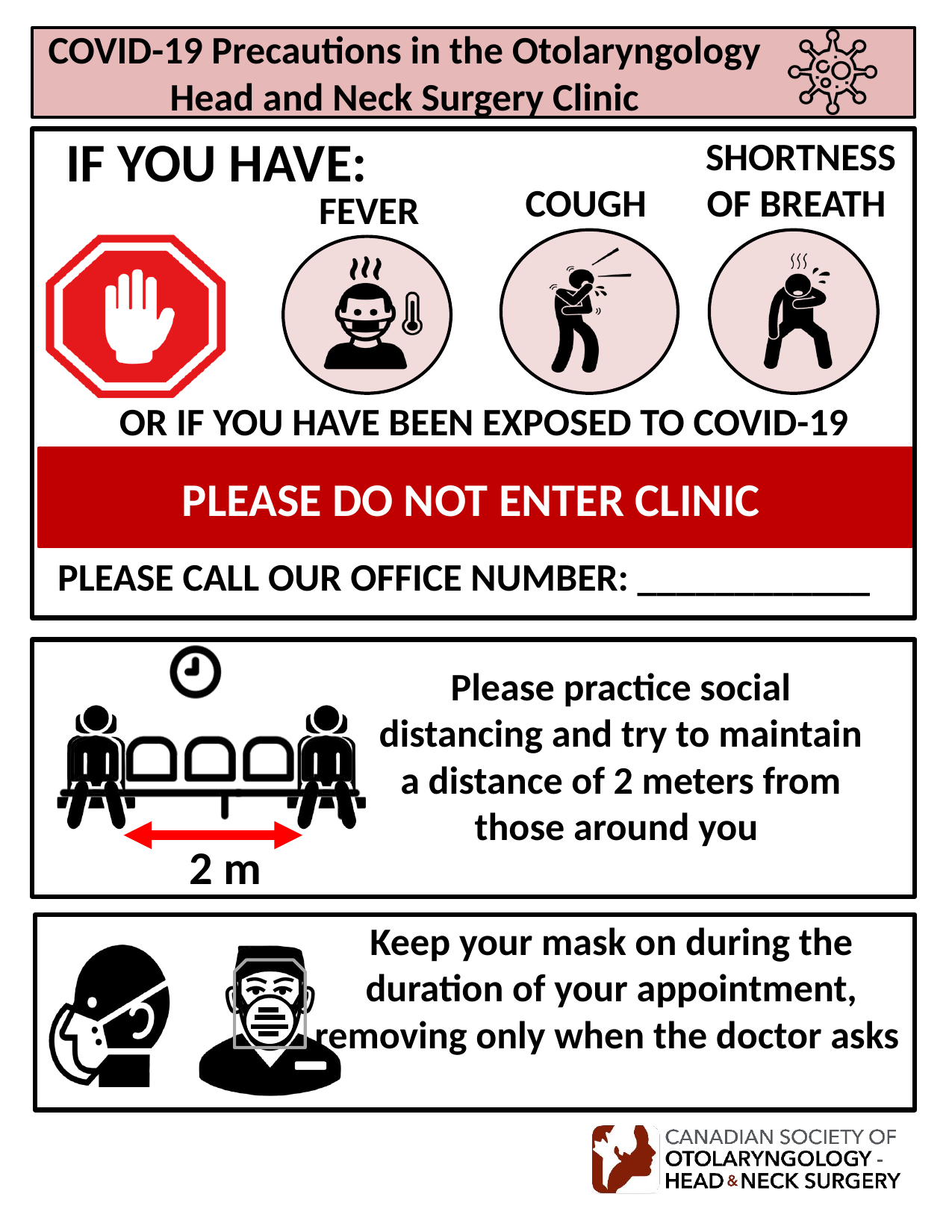

COVID-19 Precautions in the Otolaryngology
Head and Neck Surgery Clinic
IF YOU HAVE:
SHORTNESS OF BREATH
COUGH
FEVER
OR IF YOU HAVE BEEN EXPOSED TO COVID-19
PLEASE DO NOT ENTER CLINIC
PLEASE CALL OUR OFFICE NUMBER: ____________
2 m
Please practice social distancing and try to maintain a distance of 2 meters from those around you
Keep your mask on during the duration of your appointment, removing only when the doctor asks

## Slide 2
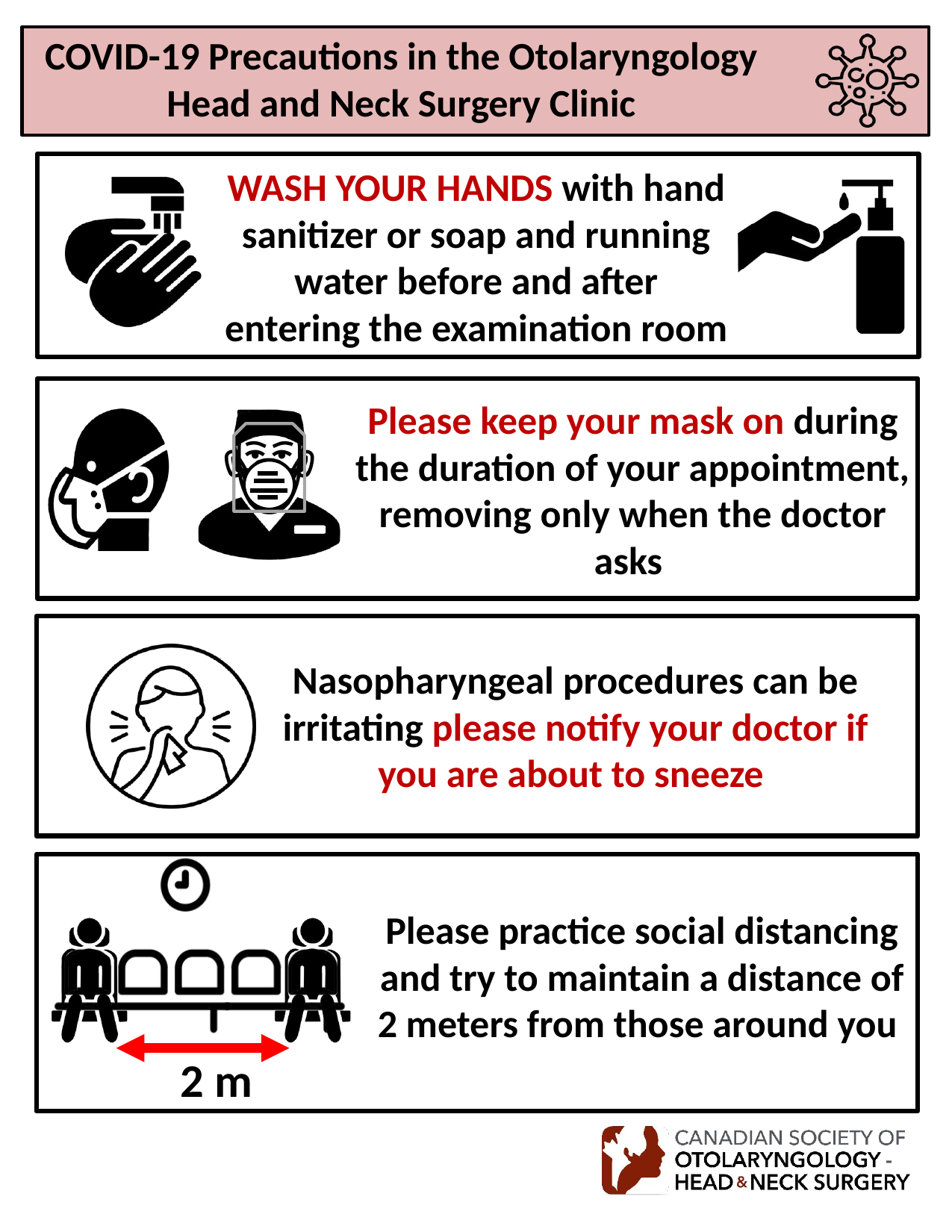

COVID-19 Precautions in the Otolaryngology
Head and Neck Surgery Clinic
WASH YOUR HANDS with hand sanitizer or soap and running water before and after entering the examination room
Please keep your mask on during the duration of your appointment, removing only when the doctor asks
Nasopharyngeal procedures can be irritating please notify your doctor if you are about to sneeze
2 m
Please practice social distancing and try to maintain a distance of 2 meters from those around you

## Slide 3
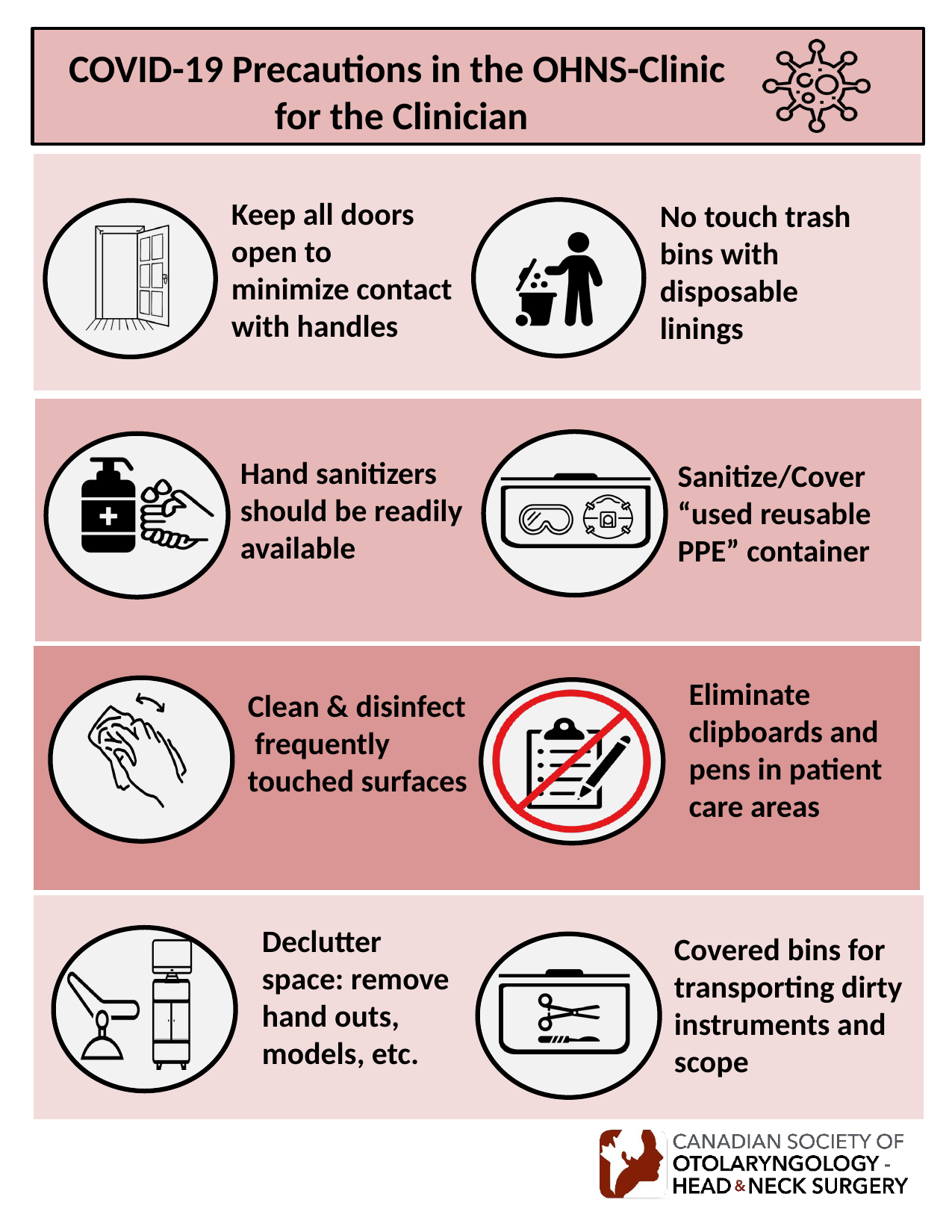

COVID-19 Precautions in the OHNS-Clinic
for the Clinician
Keep all doors open to minimize contact with handles
No touch trash bins with disposable linings
Keep all doors open to minimize contact with door handles
No touch trash bins with disposable linings
Hand sanitizers should be readily available
Hand sanitizers should be readily available
Sanitize/Cover “used reusable PPE” container
Clean and disinfect
 frequently touched surfaces
Eliminate clipboards and pens in patient care areas
Clean & disinfect
 frequently touched surfaces
Declutter space: remove hand outs, models, etc.
Declutter space: remove hand outs, models, etc.
Covered bins for transporting dirty instruments and scope

## Slide 4
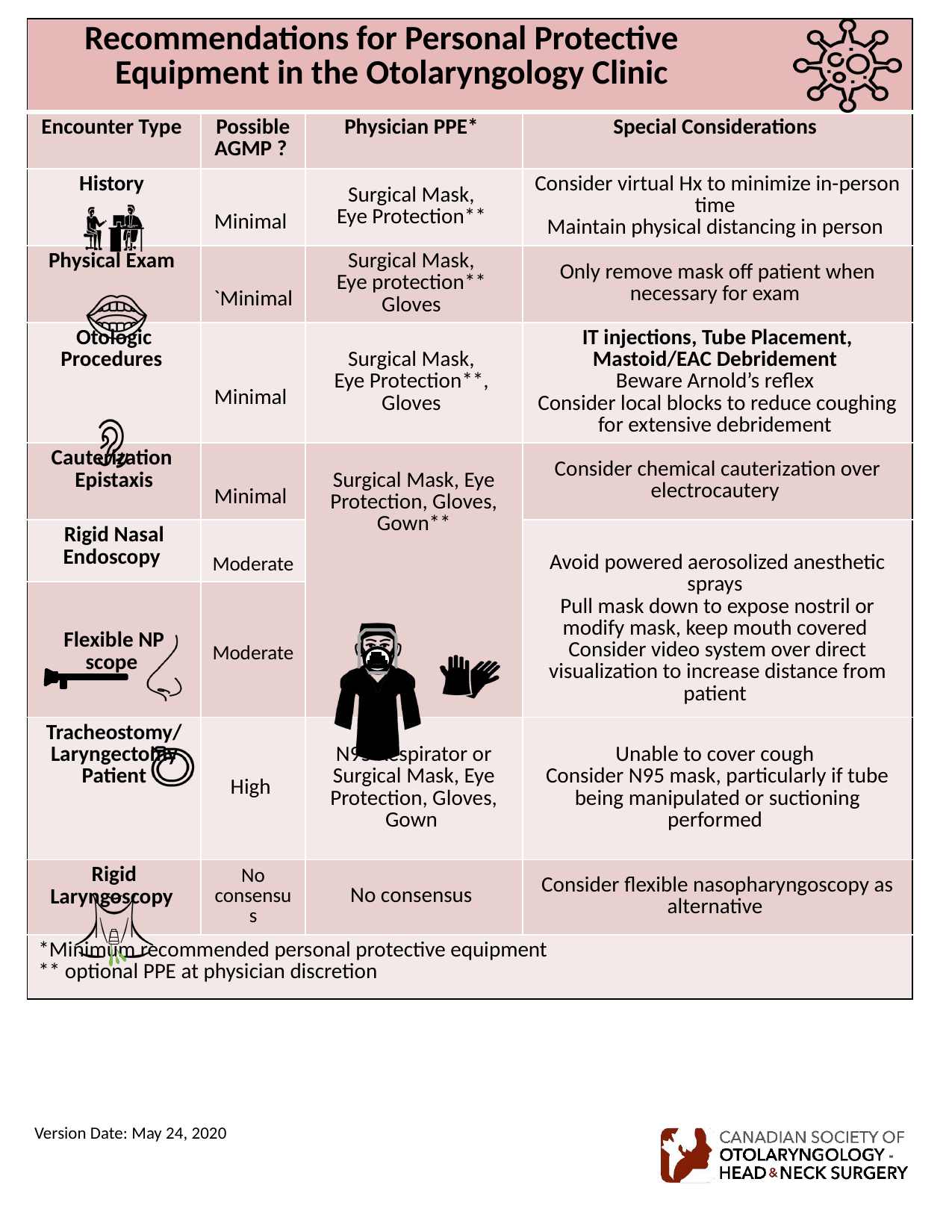

| Recommendations for Personal Protective Equipment in the Otolaryngology Clinic | | | |
| --- | --- | --- | --- |
| Encounter Type | Possible AGMP ? | Physician PPE\* | Special Considerations |
| History | Minimal | Surgical Mask, Eye Protection\*\* | Consider virtual Hx to minimize in-person time Maintain physical distancing in person |
| Physical Exam | `Minimal | Surgical Mask, Eye protection\*\* Gloves | Only remove mask off patient when necessary for exam |
| Otologic Procedures | Minimal | Surgical Mask, Eye Protection\*\*, Gloves | IT injections, Tube Placement, Mastoid/EAC Debridement Beware Arnold’s reflex Consider local blocks to reduce coughing for extensive debridement |
| Cauterization Epistaxis | Minimal | Surgical Mask, Eye Protection, Gloves, Gown\*\* | Consider chemical cauterization over electrocautery |
| Rigid Nasal Endoscopy | Moderate | | Avoid powered aerosolized anesthetic sprays Pull mask down to expose nostril or modify mask, keep mouth covered Consider video system over direct visualization to increase distance from patient |
| Flexible NP scope | Moderate | | |
| Tracheostomy/ Laryngectomy Patient | High | N95 Respirator or Surgical Mask, Eye Protection, Gloves, Gown | Unable to cover cough Consider N95 mask, particularly if tube being manipulated or suctioning performed |
| Rigid Laryngoscopy | No consensus | No consensus | Consider flexible nasopharyngoscopy as alternative |
| \*Minimum recommended personal protective equipment \*\* optional PPE at physician discretion | | | |
Version Date: May 24, 2020
